# The role of targeted next-generation sequencing and ultrasound in diagnosing fetal cytomegalovirus infection: a case report

**DOI:** 10.3389/fped.2026.1734139

**Published:** 2026-04-29

**Authors:** Jing Sun, Wenjuan Gao, Huayun Tan

**Affiliations:** 1 Obstetrics Medical Center, Weifang People’s Hospital, Shandong Second Medical University, Weifang, Shandong, China; 2 Department of Ultrasound, Weifang People’s Hospital, Shandong Second Medical University, Weifang, China

**Keywords:** cytomegalovirus (CMV), fetal, metagenomic next-generation sequencing (mNGS), polymerase chain reaction (PCR), targeted next-generation sequencing (TNGS), ultrasound

## Abstract

**Background:**

Cytomegalovirus (CMV) infection is a leading cause of congenital infection and neonatal morbidity. Conventional diagnostic methods, such as polymerase chain reaction (PCR) and amniocentesis, remain important in the diagnosis of congenital CMV infection, although each method has its own limitations in clinical practice.

**Case presentation:**

A 31-year-old woman, gravida 3 para 1, presented for routine prenatal evaluation. At 18 weeks of gestation, ultrasound revealed echogenic bowel and fetal ascites. Amniocentesis at 19 weeks showed normal chromosomal results, but targeted next-generation sequencing (tNGS) detected CMV DNA with a high viral load, confirming intrauterine infection.

**Results:**

Despite counseling regarding poor fetal prognosis, the patient chose to continue the pregnancy under close ultrasound surveillance. Progressive hydrops fetalis was observed at 23 weeks, and the pregnancy was terminated at 24 weeks.

**Conclusion:**

This case suggests that combining tNGS with ultrasound may provide complementary diagnostic information in selected cases of suspected fetal infection. In this patient, tNGS supported the identification of CMV in amniotic fluid when conventional genetic testing was unremarkable. However, as this is a single-case report, the broader diagnostic performance and clinical utility of tNGS require further validation in larger studies.

## 1 Introduction

Cytomegalovirus (CMV) is the most common congenital viral infection, affecting a significant proportion of neonates worldwide (1). Primary maternal CMV infection is an important risk factor for fetal infection and may lead to adverse outcomes such as hearing loss and neurodevelopmental impairment (2–4). Primary maternal CMV infection, particularly in early gestation, is also associated with vertical transmission and fetal sequelae (5, 6).

Fetal non-immune hydrops should raise strong clinical suspicion of intrauterine CMV infection. Typical ultrasonographic findings include generalized edema, ventriculomegaly, microcephaly, periventricular calcifications, hepatosplenomegaly, and placental thickening; however, these features are nonspecific and often overlap with other congenital infections or genetic syndromes (4, 7).

Definitive diagnosis therefore relies on virological and molecular testing. Traditional approaches such as amniocentesis, polymerase chain reaction (PCR), and serologic assays provide valuable diagnostic information in the evaluation of congenital CMV infection (8). Recent advances in molecular diagnostics have introduced targeted next-generation sequencing (tNGS) as a panel-based tool for pathogen detection that can simultaneously identify multiple predefined pathogens in a single assay (9).

Here, we report a case of congenital CMV infection evaluated through the integration of prenatal ultrasonography and tNGS.

## 2 Case presentation

A 31-year-old woman, gravida 3 para 1, with a last menstrual period dated April 18, 2025, received regular prenatal care throughout the pregnancy. Her obstetric history included one inevitable miscarriage at 22 weeks in 2020 and a cesarean delivery at 37 weeks and 4 days in 2021 for breech presentation, resulting in a healthy male infant. She had no history of hypertension, diabetes, cardiovascular disease, hepatitis, tuberculosis, or other infectious diseases.

At 8 weeks of gestation, the patient experienced mild dark-red vaginal bleeding without abdominal pain. Transvaginal ultrasound revealed no abnormalities, and no specific treatment was administered. Fetal nuchal translucency (NT) screening at 12 weeks was normal.

**Figure 1:**
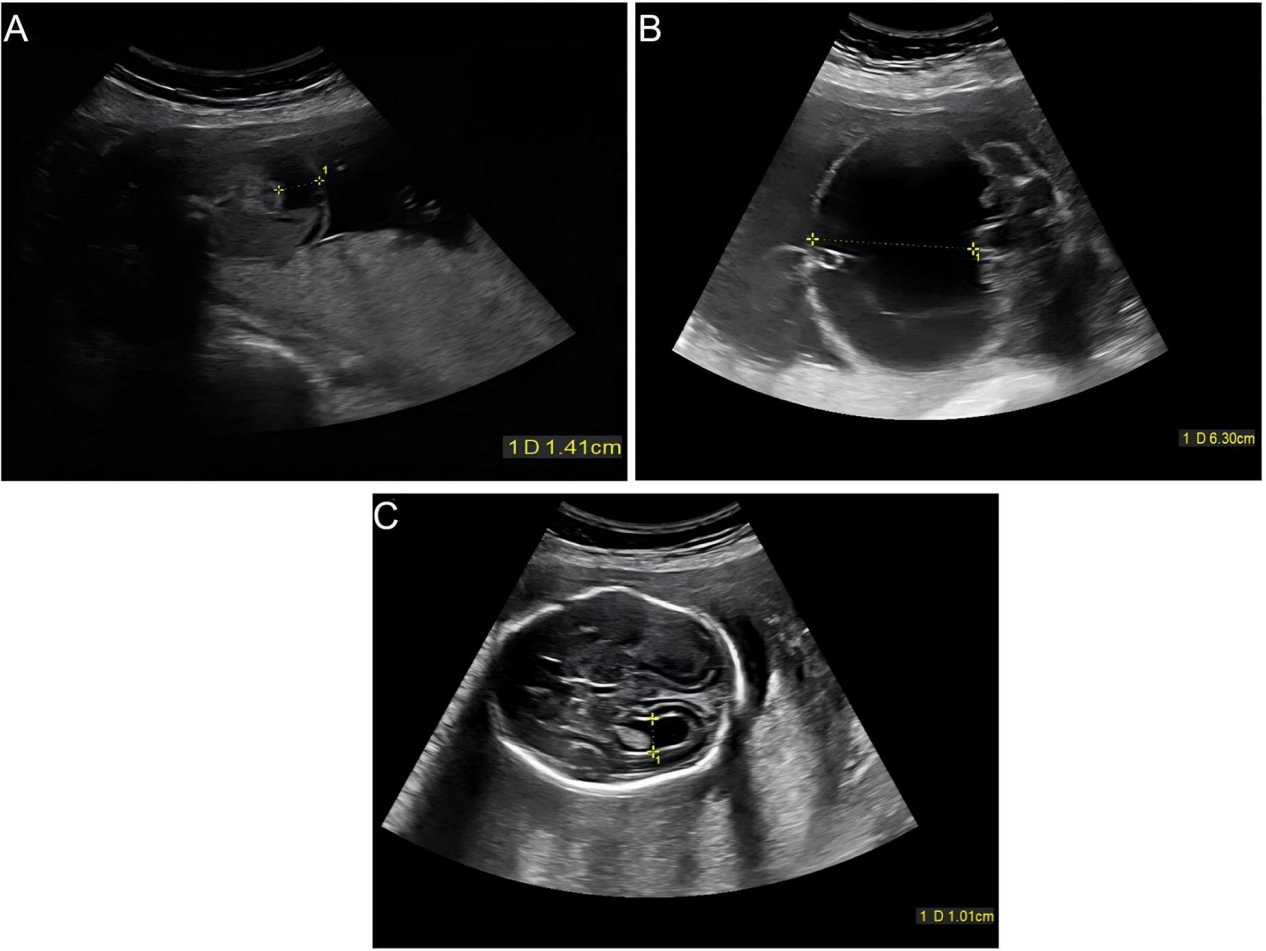
Serial prenatal ultrasound findings of congenital cytomegalovirus infection at 18 and 23 weeks of gestation. **(A)** Transabdominal ultrasound at 18 weeks showing echogenic bowel and mild fetal ascites (maximum depth, 1.4 cm). **(B)** Follow-up ultrasound at 23 weeks showing severe fetal ascites (maximum depth, 6.3 cm). **(C)** Ultrasound at 23 weeks showing ventriculomegaly, with the right lateral ventricle measuring 1.0 cm.

At 18 weeks, routine ultrasound revealed multiple abnormal ultrasound findings. The fetal bowel appeared markedly echogenic, with brightness comparable to bone, and a small intra-abdominal anechoic area was noted, measuring up to 1.4 cm in depth (Figure 1A). The fetal chest circumference was approximately 13.5 cm, and the cardiothoracic ratio was 0.53. These findings suggested a risk of chromosomal abnormality or intrauterine infection, and amniocentesis was recommended for further evaluation.

At 19 weeks of gestation, amniocentesis showed normal results on chromosomal microarray analysis (CMA) and karyotyping. In parallel, tNGS of the amniotic fluid was performed using a standardized laboratory workflow. Microbial total nucleic acids were extracted using a commercial nucleic acid extraction/purification kit (C1002, Shengwei Shuzhi, Shanghai, China) on an automated nucleic acid extractor (Natch 32A, Sansure Biotech, China). The extraction procedure was based on magnetic bead adsorption of microbial nucleic acids after lysis and digestion, followed by multiple washing steps and elution.

For library preparation, 10 μL of extracted DNA and 5 μL of reverse-transcribed RNA product were used, with a total input volume of 15 μL for targeted multiplex PCR amplification. Library construction was performed using a pathogen-targeted gene detection kit covering 296 microorganisms (Shengwei Shuzhi, Shanghai, China). Reverse transcription was first carried out using 10 μL of total nucleic acids at 25 °C for 10 min, 55 °C for 15 min, and 85 °C for enzyme inactivation. Subsequently, 5 μL of the reverse transcription product and 10 μL of total extracted nucleic acid were subjected to multiplex PCR amplification. The reaction system contained conserved sequence primers for target microorganisms (8–10 primer pairs per microorganism) as well as internal control genes for process monitoring. The amplification conditions were as follows: initial denaturation at 95 °C for 3 min; 23 cycles of 95 °C for 20 s, 63 °C for 2 min, and 72 °C for 2 min; followed by a final extension at 72 °C for 5 min.

After amplification, PCR products were purified using magnetic beads to remove residual dNTPs and impurities. Indexed library construction was then performed by adding 1 μL each of i5 and i7 index primers, followed by PCR under the following conditions: 95 °C for 3 min; 10 cycles of 95 °C for 15 s, 58 °C for 15 s, and 72 °C for 1 min; and a final extension at 72 °C for 5 min. The final libraries were purified again with magnetic beads and quantified using the Equalbit 1 × dsDNA HS Assay Kit (Vazyme, China), with 199 μL working solution plus 2 μL library DNA. Libraries were considered qualified when the fragment size was approximately 350 bp and the concentration was ≥1 ng/μL.

Qualified sub-libraries were pooled according to data requirements, diluted to 4 nM, denatured with the sequencing reagent kit, and sequenced using a single-end 50-cycle strategy based on sequencing-by-synthesis (SBS). After sequencing, raw FASTA data were demultiplexed on the instrument and subjected to downstream analysis.

Bioinformatic analysis was performed using fastp (v1.0.1) and BWA (v0.7.19) for quality control, adapter trimming, and sequence alignment. Pathogen identification and annotation were completed using an in-house curated database derived from RefSeq and GenBank after deduplication, together with supporting scripts. Background noise was removed using in-house denoising scripts. Final results were integrated and interpreted using an integrated bioinformatic analysis system (Sansure Biotech), which generated a standardized clinical report.

tNGS detected CMV DNA, with a normalized sequence count of 237,162 and an estimated viral concentration of 6 × 10^5^ copies/mL, confirming intrauterine CMV infection. The patient received detailed counseling regarding the high risk of adverse fetal outcomes and, after comprehensive informed discussion, chose to continue the pregnancy under close ultrasound surveillance.

Follow-up ultrasound at 23 weeks demonstrated marked disease progression. The fetal abdomen was distended with increased ascitic fluid (maximum depth 6.3 cm) (Figure 1B). The right lateral ventricle measured 1.0 cm (Figure 1C), and the placenta was thickened to 6.9 cm with heterogeneous echotexture. The amniotic fluid index had increased to 10 cm. Fetal biometric parameters were as follows: biparietal diameter 6.2 cm and abdominal circumference 30.0 cm. These findings indicated progressive ascites, ventriculomegaly, polyhydramnios, and secondary pulmonary hypoplasia, consistent with hydrops fetalis.

After multidisciplinary discussion and comprehensive prenatal counseling, the patient elected to terminate the pregnancy at 24 weeks due to the extremely poor fetal prognosis.

On admission, vital signs were stable: temperature 36.5 °C, pulse 100 beats/min, respiratory rate 18 breaths/min, and blood pressure 120/73 mmHg. Cardiorespiratory examination was unremarkable. Obstetric examination revealed a markedly distended abdomen (fundal height 29 cm, abdominal circumference 105 cm), larger than expected for gestational age. Laboratory results showed a white blood cell count of 10.05 × 10⁹/L (reference 3.5–9.5 × 10⁹/L), lymphocytes 13.9% (reference 20%–50%), neutrophils 77.6% (reference 40%–75%), and hemoglobin 104 g/L (reference 115–150 g/L); other parameters were within normal limits.

Labor induction was performed via intra-amniotic injection of ethacridine lactate (100 mg). Two days later, uterine contractions commenced, and a male stillborn was delivered vaginally. The fetus exhibited marked abdominal distension. The placenta was enlarged, friable, and partially adherent to the uterine wall. Manual removal resulted in significant bleeding, which was controlled with uterotonic and hemostatic agents. Residual placental tissue was subsequently removed under ultrasound guidance.

Placental histopathology revealed extensive infiltration of lymphocytes and neutrophils with focal abscess formation, consistent with chorioamnionitis. The patient received postoperative antibiotic and uterotonic therapy, recovered well, and was discharged in stable condition on the third day after delivery. Follow-up confirmed satisfactory maternal recovery.

Serological testing for maternal CMV infection was not systematically performed in this case; therefore, it could not be determined whether the maternal infection represented primary infection, viral reactivation, or reinfection with a new viral strain. In addition, maternal blood CMV viral load was not quantified.

## 3 Discussion

CMV infection during pregnancy may result in intrauterine transmission, and fetal manifestations can vary depending on maternal and fetal factors (10). Typical ultrasound manifestations of fetal CMV infection include echogenic bowel, ventriculomegaly, intracranial calcifications, periventricular halo, ascites, hepatosplenomegaly, placentomegaly, and, in severe cases, hydrops fetalis or cerebral hemorrhage (4, 7, 11). In this case, the patient presented with fetal echogenic bowel and ascites at 18 weeks of gestation, with the echogenicity of the bowel similar to that of bone, a typical ultrasound finding for CMV infection (11). As the pregnancy progressed, at 23 weeks, the fetal ascites worsened (maximum depth of 6.3 cm), accompanied by an increased cardiothoracic ratio (0.53) and a thickened placenta (6.9 cm), suggesting disease progression and consistent with ultrasound findings of hydrops fetalis due to CMV infection (7). However, intracranial calcifications were not observed in this case, with only ventricular enlargement present, which differs from some reported cases of more severe CMV infections (7, 11).

Ultrasound findings for fetal CMV infection lack specificity, so in cases of unexplained fetal hydrops with abnormal ultrasound, amniocentesis is needed to confirm CMV infection and exclude chromosomal abnormalities (2, 7, 12).

Conventional diagnostic methods, including CMV qPCR and maternal serologic testing, remain important in the diagnosis of fetal CMV infection. Different diagnostic methods provide different types of information and should be interpreted together with ultrasound findings and the overall clinical context (3, 10, 12, 13). tNGS may offer practical value in the diagnosis of infectious diseases (9, 14). As a panel-based sequencing method, tNGS enables the simultaneous detection of multiple predefined pathogens, including bacteria, viruses, fungi, and parasites, within a single assay (9). In the present case, tNGS identified CMV in amniotic fluid in the context of abnormal fetal ultrasound findings.

The interpretation of tNGS results in this study was based on normalized sequence counts, background signal removal, and consistency with the clinical presentation. Positive findings were defined by reads exceeding background noise after bioinformatic denoising and by alignment to the curated reference database. Low-level microbial reads were interpreted cautiously, taking into account the possibility of environmental contamination, nonspecific amplification, and sequencing artifacts. Therefore, tNGS results were not interpreted in isolation but in combination with fetal ultrasound abnormalities, invasive prenatal testing results, and the overall clinical context. In this case, the high normalized CMV sequence count and estimated viral concentration supported the diagnosis of clinically significant intrauterine CMV infection.

In this report, tNGS was interpreted as a complementary method for predefined pathogen screening when broader testing was clinically considered. However, this study is a single-case report, and no formal diagnostic thresholds, sensitivity, specificity, positive predictive value, or negative predictive value could be established. These performance parameters should be assessed in future studies with larger sample sizes.

Compared with conventional targeted molecular methods, tNGS allows simultaneous detection of multiple predefined pathogens in a single assay. In addition, although tNGS may offer advantages in detecting known pathogens and supporting clinical decision-making, its limitations include the inability to identify pathogens beyond the established panel, as well as relatively high technical requirements and cost (9). However, because it depends on a predefined target panel, it is not suitable for detecting novel or unexpected pathogens outside the panel design. Therefore, tNGS should be regarded as a complementary diagnostic tool rather than a replacement for all existing methods.

In this case, amniocentesis at 19 weeks revealed normal results through chromosomal microarray analysis (CMA) and fetal karyotyping, while tNGS detected CMV DNA in amniotic fluid, supporting the diagnosis of intrauterine CMV infection. This case does not demonstrate formal diagnostic superiority over PCR or other conventional methods; rather, it suggests the potential complementary value of tNGS in situations where broader predefined pathogen screening may be clinically useful.

The patient was diagnosed with intrauterine CMV infection at 19 weeks using tNGS. Due to the lack of effective antiviral treatment for CMV, despite the potential risks, the patient chose to continue the pregnancy at 19 weeks with close ultrasound monitoring (13). However, due to dynamic ultrasound findings at 23 weeks, showing a significant increase in fetal ascites and fetal hydrops, indicating poor prognosis, the patient, after thorough consideration, decided to terminate the pregnancy at 24 weeks. The stillborn fetus exhibited severe ascites and noticeable hydrops, further supporting the decision to terminate the pregnancy.

This case suggests that tNGS may serve as a complementary diagnostic tool in selected cases of suspected fetal infection, but its limitations include the inability to detect pathogens outside the established panel, as well as high costs and technical requirements (8, 15). However, when combined with ultrasound, it may provide complementary diagnostic information that supports clinical decision-making.

## 4 Conclusion

This case suggests that combining tNGS with ultrasound may provide complementary diagnostic information in selected cases of suspected fetal infection. In this patient, tNGS supported the identification of CMV in amniotic fluid when conventional genetic testing was unremarkable. However, as this is a single-case report, the broader diagnostic performance and clinical utility of tNGS require further validation in larger studies.

## 5 Patient perspective

The patient appreciated that the diagnostic evaluation helped clarify the fetal condition and guide decision-making.

## Data Availability

The original contributions presented in the study are included in the article/Supplementary Material, further inquiries can be directed to the corresponding authors.
